# Are Shopkeepers Suffering from Pulmonary Tuberculosis in Bahir Dar City, Northwest Ethiopia:* A Cross-Sectional Survey*

**DOI:** 10.1155/2017/2569598

**Published:** 2017-12-07

**Authors:** Mulusew Andualem Asemahagn

**Affiliations:** School of Public Health, College of Medicine and Health Sciences, Bahir Dar University, Bahir Dar, Ethiopia

## Abstract

**Background:**

Despite several interventions, tuberculosis (TB) continues to be a major public health concern in developing countries.

**Objective:**

To determine pulmonary TB prevalence and associated factors among shopkeepers in Bahir Dar City, Ethiopia.

**Methods:**

A cross-sectional study was conducted in 2016 among 520 shopkeepers who had TB signs and symptoms using questionnaire interview and sputum samples processing. Shopkeepers were considered TB positive if two sputum slides became positive. Data were edited and analyzed using SPSS version 23. Multivariable logistic regression analysis was used to identify factors.

**Results:**

A total of 520 shopkeepers were interviewed and gave sputum samples. About 256 (49.2%) of them were under the ≤30 years' age category, 22.0% can read and write, 65.0% were Muslims, and 32.0% originated from rural areas. Pulmonary TB prevalence was 7.0% (37/520), and positivity proportion was 57.0% (21/37) in males and 70.0% (26/37) in urban residents. Smaller (44.0%) shopkeepers got health education on TB. Illiteracy, no health education, contact history, cigarette smoking, nonventilated shops, and comorbidities were factors to TB infection (*p* value < 0.05).

**Conclusions:**

Significant numbers of shopkeepers were infected by TB. Factors to TB infection were either personal or related to comorbidities or the environment. Therefore, TB officials need to specially emphasize awareness creation, occupational health, and early screening to prevent TB.

## 1. Introduction

Despite several international and national anti-TB interventions, TB continues to be the ninth leading cause of global morbidity and the leading cause of a single infectious agent, ranking above HIV/AIDS [1]. TB is the first killer contagious disease among people living with HIV/AIDS [1, 2]. Based on the WHO 2017 report, there were 10.4 million new TB cases: 90% adults, 65% men, and 10% people living with HIV/AIDS (74% from Africa). There were also about 1.3 million global TB deaths among HIV negatives and 374 000 deaths among HIV positives in 2016. Similarly, five countries (India, Indonesia, China, the Philippines, and Pakistan) accounted for 56% of the global TB incidence cases [1].

The 2017 WHO report also showed varied distribution of TB incidence cases across the WHO regions in 2016: 45% of TB cases occurred in the WHO Southeast Asia Region and 25% in the WHO African Region followed by the 17% in the WHO Western Pacific Region [1]. Likewise, Nigeria and South Africa each accounted for 4% of the global total. Poor socioeconomy, high HIV/AIDS burden, malnutrition, poor community awareness, poor healthcare services, and low TB case detection could be potential explanations for high TB incidence in the sub-Saharan Africa [1–6].

Although several anti-TB interventions have been made in Ethiopia, the country continues to be the 7th country in TB incidence among the 22 high TB burden countries globally and 4th from Africa. Ethiopia is also among the top 20 countries with TB-HIV and drug resistance TB (MDR-TB) from the 30 high TB-HIV and MDR-TB burden countries [1, 2]. According to the Ethiopian Federal Ministry of Health hospital statistics evidence, pulmonary TB was the third leading cause of hospital admission (7.8%) and the first leading cause of in-patient deaths (10.1%) [7]. Even though the Ethiopian government has expanded TB diagnosis and treatment services to both the public and private healthcare facilities since 2004, TB is still major public health concern. This may be due to the emergency of drug resistance TB, malnutrition, low community awareness, inaccessibility/quality of TB services, and high comorbidity rates (HIV/AIDS, malaria, and diabetes mellitus) [1–12].

Shopkeepers, people who serve as seller in any shop either for a full or part time, are assumed high risk population groups to TB infection as a result of double infection sources: frequent contact with different customers whose health status is unknown and family or community sources. In addition, overcrowdedness of shops and absence of occupational health safeties such as access to healthcare services including early screening to TB, HIV/AIDS, and others and health insurance may aggravate the risk of acquiring TB infection shopkeepers, mainly in the developing countries. Hence, shopkeepers not only are among the high risk population groups to acquire TB infection but are also high source of TB infection to their families, friends, and the rest of the community.

Although shopkeepers are at high risk of getting TB infection and there are several types of shops where shopkeepers are working in Ethiopia, almost all do not get special attention on TB prevention from the healthcare facilities and TB control program. This may contribute more for the presence of high TB infection burden in Ethiopia. According to my knowledge and search, there is no study conducted on TB infection among shopkeepers in Africa and Ethiopia.

Therefore, this study was aimed at determining the prevalence of pulmonary TB infection and identifying factors associated with TB infection among shopkeepers in Bahir Dar City, Northwest Ethiopia. This will serve as crucial evidence to Ethiopian Federal Ministry of Health, Amhara Regional Health Bureau, Bahir Dar City administration health office, and the nongovernmental organizations working on TB prevention to know the burden of TB infection on shopkeepers and identify associated risk factors so that they will have evidence based intervention plan. It will also be important source to the coming researchers who are interested in TB prevention.

## 2. Materials and Methods

### 2.1. Study Design and Area

A cross-sectional study was conducted between October and December 2016 among shopkeepers in Bahir Dar City, Northwest Ethiopia. Bahir Dar City is the capital city of Amhara Regional State and located 565 kilometers to the Northwest of Addis Ababa, the capital city of Ethiopia. The city has nine administrative subcities, two public hospitals, and six public health centers. The Amhara Regional Health Bureau and Regional Public Health Institute are found in the city. More than 2400 legally recognized shops were found in the city. Majority of the shops are located at the center of the city, “Tana Market center” and around it [9].

### 2.2. Sample Size Determination and Technique

Due to time, feasibility, and resource constraints, only one-third of the total shops (800 out of 2400) were included in the study through simple random sampling technique by taking the sampling frame from the Amhara Region trade office. After assessing the presence of signs and symptoms including presence of cough for 14 days and above, only 520 out of 1600 shopkeepers working in 800 shops were included in the study.

### 2.3. Data Collection Tools and Procedures

#### 2.3.1. Sociodemographic Data and Risk Factors Assessment

Data on the sociodemographic and associated risk factors were collected using pretested structured questionnaire. Three bachelor nurses collected data through interviewer-administered approach. The questionnaire was comprised of data on sociodemographic data (age, sex, education level, family size, income level, religion, residence, etc.) and risk assessment on TB infection (crowdedness, previous TB contact, awareness/health education access, room size, comorbidity, personal behavior/smoking, khat chewing, alcohol intake, and cough duration). Shopkeepers who either smoke or drink alcohol or chew khat in any amount in a daily manner were considered as poor personal behavior since they have a direct or indirect linkage to TB infection. Awareness of study participants was assessed via access to health education by healthcare workers or media on TB transmission, risk factors, signs and symptoms, and prevention mechanisms. Those who got health education on TB at least one time were considered as having awareness on TB transmission and prevention. The ventilation and crowdedness of the shops were estimated by observation. On the other hand, data on shops' room size were collected through measuring the size by a meter scale.

#### 2.3.2. Sputum Samples Collection, Processing, and Examination

Three laboratory technicians collected sputum data. They informed interviewed shopkeepers how to collect sputum samples and gave them clean, labeled, and dry tightly caped sputum caps to bring samples. Study participants provided three sputum samples (spot-morning-spot) for Acid-Fast staining. Sputum sample collection, smear preparation, AFB staining, and smear examination were done according to the Ethiopian National TB and Leprosy prevention and control standard guideline [3]. Then, stained smears were examined through Olympus microscope and shopkeepers were grouped under TB positives if at least two AFB sputum smear slides were positive.

### 2.4. Data Quality Assurance and Analysis

Training of data collectors, questionnaire validation, providing information on sample collection, labeling sputum caps/slides, running quality control slides parallel to the sputum samples, rereading of all positive sputum slides and 15% of the negative slides by the experienced laboratory technicians, and following the national guideline in all steps were major quality assurance activities. Data were edited and analyzed using the SPSS version 22 software. Different descriptive statistics (percentages, proportions, count, etc.) and binary logistic regression analyses were computed to describe study objectives and identify factors associated with TB infection among shopkeepers, respectively. Odds ratio at 95% confidence level was used to describe statistical association between the study and outcome variables.

### 2.5. Ethical Consideration

The study was ethically cleared and approved by the ethical review committee of Amhara Regional Health Bureau. A support letter was taken from the Bahir Dar City administration Health office after discussing the purpose and data collection procedures of the study. Shop owners and study participants were informed on the study objective, data collection procedures, and data confidentiality issues prior to the data collection. Informed consent was taken from each shopkeeper and participation was fully voluntary. Data confidentiality was kept through anonymity. TB positive shopkeepers were counseled and linked to the nearby health facilities and started their anti-TB treatment according to the Ethiopian national TB treatment guideline.

## 3. Results

### 3.1. Sociodemographic Characteristics of the Participants

A total of 520 shopkeepers (224 males and 296 females) participated in this study. Nearly half, 256 (49.2%), of the shopkeepers were under the age category of ≤30 years. The mean age and standard deviation of the shopkeepers were 30 ± 5 years. Large number of respondents (61.0%) were married in marital status. About two-thirds (65.0%) of the shopkeepers were Muslims and 32.0% originated from the rural areas. Only 12.0% of the shopkeepers cannot read and write and 42.0% had contact history with known TB cases. Less than half (44.0%) of shopkeepers got health education on TB infection from health extension workers. More than half (54.0%) of them had ≤5 years of work experience as shopkeeper. Nearly three-fourths (70.0%) of the shops were nonventilated/crowded by goods and there was no adequate space/window for air circulation. Over two-thirds (68.0%) of the shops had ≥4 square meter area size (Table 1).

Personal behavior of shopkeepers was described in terms of alcohol intake (any type), smoking, and khat chewing in any amount daily. Over a third, 191 (36.7%), of the shopkeepers had history of alcohol intake of any type daily. Likewise, 222 (42.7%) and 203 (39.0%) of them were cigarette smokers and khat chewers daily, respectively. Regarding comorbidity history, 182 (35%) of the shopkeepers had history of HIV/AIDS and/or DM (Table 2).

### 3.2. Results of Sputum Microscopy

The prevalence of smear positive pulmonary TB among shopkeepers who had cough for 14 days and above was 7.0% (37/520); 4.0% (21/520) males; and 3.0% (16/520) females. The proportion of TB positivity rate was higher among shopkeepers with age category of ≤30 years: 62.2% (23/37). Similarly, the proportion of TB positivity rate was higher among male, Muslims, urban residents, married, and smokers and from nonventilated shop respondents. Only 10% of the respondents did TB screening before this study. Over a third (35.0%) of the shopkeepers had comorbidity history such as HIV/AIDS and/or diabetes mellitus (DM) (Table 2).

### 3.3. Risk Factors Associated with TB Infection of Shopkeepers

Education level (illiterate), cigarette smoking, having contact with TB patients, not getting health education on TB, having comorbidity (HIV/AIDS and/or DM), and being in poorly ventilated shops were found to be statistically significant risk factors to acquire TB infection among shopkeepers (*p* value < 0.05). The odds of acquiring TB infection were double times among illiterate shopkeepers compared to shopkeepers who can read and write (OR = 2.14, 95% CI = [1.21–4.75], *p* value = 0.004). Similarly, TB infection was found higher among shopkeepers who had contact history with confirmed TB cases compared to their counterparts (OR = 1.85, 95% CI = [1.16–3.69], *p* value = 0.010). Shopkeepers who had cigarette smoking habits were twice in acquiring TB infection than nonsmoker shopkeepers; OR = 2.16, 95% CI = [1.14–3.45], *p* value = 0.012. Likewise, the odds of getting TB infection were 64% times less likely among shopkeepers who got health education on TB than those who did not get health education (AOR = 0.36, 95% CI = [0.16–0.62], *p* value = 0.030). Also, nonventilated shops were found to be determinant factor in getting TB infection among shopkeepers; those who worked inside the ventilated shops were 70% times less likely to acquire TB infection than the respective groups (OR = 0.30, 95% CI = 0.11–0.58, *p* value = 0.023). Shopkeepers with HIV/AIDS and/or DM were 1.96 times more likely to be TB positive compared to shopkeepers with no comorbidity history (OR = 1.96, 95% CI = [1.14–3.22], *p* value = 0.012) (Table 2).

## 4. Discussion

The current study revealed that pulmonary TB prevalence is important health problem among shopkeepers in Bahir Dar City (7.0%: 7 in 100). This clearly indicates that shopkeepers are at the high risk of getting TB infection, because they have double infection sources: from the customers and families/community. It means that they have high chance of getting conformed and nonconfirmed TB cases without taking care to prevent them from getting the infection. In addition, education level (illiteracy), working inside the nonventilated rooms, low awareness of TB transmission, and comorbidity with other chronic diseases such as HIV/AIDS and DM were statistically significant factors to acquire TB infection among shopkeepers (Table 2). All these were reported challenges to the END TB program in 2015 by the WHO [2] and other studies [5–10].

The situation among shopkeepers is serious in TB transmission due to the nature of their work which will highly expose them to contacting more people per day with minimal or no precautions compared to other institutions. This makes the condition severe because they can easily get TB infection and become potential sources of infection to their families, customers, and the community at large. Hence, TB infection may continue to be the most prevalent among shopkeepers and become big challenge to the END TB program unless appropriate interventions such as continuous information provision, planning TB screening, and treatment services to the high risk areas, improving rooms' conditions, and managing other comorbidities are made. This obviously shows that TB programmers at different levels need to consider additional TB prevention strategies to address communities who are at the high risk of getting TB infection. This was also one of the recommended strategies of the END TB program by the WHO [1, 2] and Ethiopian Federal Ministry of Health [11].

Due to the absence of published evidences on TB prevalence among shopkeepers, the author tried to discuss present prevalence with studies at the community, facility, and prison levels in Ethiopia; since they have some similarities, they are relatively higher, risky/congregated settings/to acquire TB infection [13]. The current prevalence was found to be higher compared to study findings from Dessie and Debre-Birhan (among homeless participants) [5], people attending spiritual holy water services as the alternative medication to their illnesses [10], and North Gondar [12, 14] where TB prevalence was 2.6%, 2.9%, 5.3%, and 4.9%, respectively. The possible explanation for this variation could be differences in area coverage, comorbidity prevalence, information access, contact status with TB patients, and overcrowdedness. In the current study, poor room ventilation (70%), low health education on TB (44%), and high comorbidity with HIV/AIDS and DM (35%) were assisting factors to TB infection. Hence, these could be potential sources to the variation of TB prevalence compared to the studies stated above.

Consistently, the current prevalence was also higher compared to study findings from abroad: China, 2.7% [15]; India, 4/1000 [16]; and Tanzania, 3.6% [17]. It could be due to variations in personal awareness, TB programs performance, ventilation status, comorbidity, nutritional status, and overcrowdedness.

On the other hand, TB prevalence among shop keepers was found to be lower compared to study findings from Gamo Gofa zone, Ethiopia (19.4%) [18]; Nigeria (21.15%) [19]; and Brazil (25.2%) [20]. This variation could probably be attributed to differences in study period, study area coverage, study population, and methods employed. For example, culture was used to detect TB cases in the case of Gamo Gofa zone which possibly could increase positivity rate. Similarly, in Brazil, Tuberculin skin test was used in addition to sputum microscopy.

In this study, the odds of acquiring TB infection were higher among illiterate shopkeepers (OR = 2.14, 95% CI [1.21–4.75]) compared to those who can read and write. It is known that literacy level is crucial to prevent TB infection because it can determine the understanding level of individuals on TB transmission, prevention, and information searching skills. Evidences by the WHO [2] and other studies [11, 21] also revealed that education level was determinant factor to TB infection.

Correspondingly, TB prevalence was higher among shopkeepers who had cigarette smoking history than the nonsmoker participants (Table 2). This fact was in line with study findings from Ethiopia [3, 8, 11, 12] and abroad [2, 22]. The possible justification could be related to the impact of smoking on lung functionality. Smoking could affect the lung and became a predisposing factor to lung diseases and make individuals more susceptible to pulmonary TB infection.

In this study, the odd of acquiring TB infection was almost double among shopkeepers who had previous contact history with confirmed TB cases (Table 2). This was supported by several study findings [2–7, 11, 12, 22, 23]. It is because one of the primary transmission routes to TB infection is long contact/living with untreated TB cases. Therefore, shopkeepers who have longer contact with several people (customers) have the highest chance of acquiring and transmitting TB from/or to the family, neighbors, customers, and the community.

Similarly, health education was found to be preventive strategy for TB infection; shopkeepers who got health education by the healthcare workers in the past two years about TB transmission and prevention were 64% times less likely to get TB infection compared to the counterparts. The WHO also strongly recommended the importance of awareness creation, and health promotion on TB prevention [2]. It is also one of the basic strategies in Ethiopian health sector transformation plan [8]. This is because health education can facilitate health information dissemination and awareness creation among the community. Thus, they can prevent TB infection through either avoiding the predisposing factors or early diagnosis and treatment of TB infection. Hence, TB programmers in Ethiopia and Bahir Dar City need to think of planning special strategies on accessing health education to shop keepers and people in similar settings since they have lesser chance of getting health education given by the health extension workers at the household levels due to spending their time at shops, and the percentage of health education among shopkeepers was lower (44%).

Another important determinant factor to TB infection was comorbidity history of shopkeepers. The odd of getting TB infection was nearly twofold among shopkeepers who had comorbidity (HIV/AIDS and/or DM) compared to those who had no comorbidity history (Table 2). Almost all studies, reports, and guidelines supported this idea [1–8]. It is because comorbidities such as HIV/AIDS and DM can compromise human immune system and make individuals more susceptible to TB infection. Therefore, regular health education and early screening for TB, HIV, DM, and other chronic diseases are vital among high risk population groups such as shopkeepers.

In addition to the above-mentioned risk factors, shop conditions showed statistically significant association with TB infection in this study: shopkeepers working in ventilated shops were 70% times less likely to acquire TB infection than their counterparts (Table 2). The most important WHO recommendation to prevent TB infection is to have ventilated areas anywhere (working places, homes, hotels, transportation, and public service places). This is because the primary transmission route of TB infection is through air droplets and if the rooms have no two-way parallel air circulations, everyone who enter or stay there can potentially get TB infection. For this case, shops are typical examples since most (32%) were very narrow (less than 4-square meter area), overcrowded/no air circulation (Tables 1 and 2). This clearly shows that special attention is required to make shops occupationally safe to shopkeepers and customers with the emphasis on preventing them from acquiring communicable diseases, particularly TB. In addition, it is better to implement occupational health strategies in such settings to increase individuals' safety.

## 5. Conclusions and Recommendations

Based on this study, pulmonary TB is found to be an important health problem among shopkeepers (7 in 100). Being unable to read and write, cigarette smoking, not getting health education, previous contact with TB cases, comorbidity (HIV/AIDS and/or DM), and shops' condition were statistical significant factors of TB infection among shopkeepers in Bahir Dar City. Thus, special attention on awareness creation, early screening, treatment (TB, HIV/AIDS, and DM), interventions on poor personal behaviors (cigarette smoking, etc.), implementing occupational health strategies, and improving shops ventilation are crucial for preventing TB infection.

## 6. Limitations and Implications

In this study, only quantitative data and sputum microscopy were used as data collecting tools which may have limitation on identifying associated risk factors and determining the TB positivity rate among shopkeepers, respectively. Absence of literature on TB infection among shopkeepers was also another limitation to this research work.

## Figures and Tables

**Table 1:** Sociodemographic variables of shopkeepers in Bahir Dar City, Ethiopia, 2017.

Variable	Responses	Frequency	Percent (%)
Age	≤30	256	49.0
	>30	264	51.0
Sex	Male	224	43.0
	Female	296	57.0
Religion	Christians	183	35.0
	Muslims	337	65.0
Marital status	Single	203	39.0
	Married	317	61.0
Residence	Rural	167	32.0
	Urban	353	68.0
Education level	Cannot read and write	63	12.0
	Can read and write	457	88.0
Work experience	≤5 years	281	54.0
	>5 years	239	46.0
Contact with TB cases	Yes	219	42.0
	No	301	58.0
Got health education on TB	Yes	229	44.0
	No	291	56.0
Shop size in meter square	≥4	354	68.0
	<4	166	32.0
Shops' condition	Ventilated	156	30.0
	Nonventilated	364	70.0

**Table 2:** TB infection and its determinant factors among shopkeepers in Bahir Dar City, Ethiopia, 2017.

Variables	TB status		COR (95 CI)	AOR (95% CI)	*p* value
	Positive (%)	Negative (%)			
Age in years					
≤30	23 (4.4)	233 (44.8)	1.76 [0.89–3.51]	0.85 [0.27–2.64]	0.103
>30	14 (2.7)	250 (48.1)	1	1	
Sex					
Male	21 (4.0)	203 (39.0)	1.81 [0.92–3.56]	0.86 [0.17–2.41]	0.081
Female	16 (3.0)	280 (54.0)	1	1	
Marital status					
Single	15 (2.9)	188 (36.2)	1.10 [0.54–2.11]	0.46 [0.15–1.83]	0.846
Married	22 (4.2)	295 (56.7)	1	1	
Residence					
Rural	11 (2.1)	156 (30.0)	0.88 [0.43–1.84]	0.52 [0.21–1.53]	0.747
Urban	26 (5.0)	327 (62.9)	1	1	
Religion					
Christians	9 (1.7)	173 (33.3)	0.58 [0.26–1.25]	0.24 [0.14–1.20]	0.158
Muslims	28 (5.4)	310 (59.6)	1	1	
Education level					
Illiterate	10 (1.9)	53 (10.2)	3.01 [1.38–6.55]	2.14 [1.21–4.75]	0.004
Can read and write	27 (5.2)	430 (82.7)	1	1	
Alcohol intake					
Yes	17 (3.3)	174 (33.5)	1.51 [0.77–2.96]	0.89 [0.32–2.16]	0.228
No	20 (3.8)	309 (59.4)	1	1	
Cigarette smoking					
Yes	23 (4.4)	198 (38.1)	2.40 [1.19–4.71]	2.16 [1.14–3.45]	0.012
No	14 (2.7)	285 (84.8)	1	1	
Khat chewing					
Yes	19	184	1.71 [0.87–3.35]	0.75 [0.42–2.36]	0.111
No	18	299	1	1	
Contact with TB cases					
Yes	23 (4.4)	196 (37.7)	2.41 [1.21–4.79]	1.85 [1.16–3.69]	0.010
No	14 (2.7)	287 (55.2)	1	1	
Got TB Health education					
Yes	10 (1.9)	219 (42.11)	0.45 [0.21–0.94]	0.36 [0.16–0.69]	0.030
No	27 (5.2)	264 (50.80)	1	1	
Work experience					
≤5 years	16 (3.1)	265 (51.0)	0.63 [0.32–1.24]	0.41 [0.26–1.17]	0.172
>5 years	21 (4.0)	218 (41.9)	1	1	
Shops' condition					
Ventilated	5 (1.0)	151 (29.0)	0.35 [0.13–0.89]	0.30 [0.11–0.58]	0.023
Not ventilated	32 (6.1)	332 (63.9)	1	1	
Has comorbidity					
Yes	20 (3.8)	162 (31.2)	2.33 [1.19–4.57]	1.96 [1.14–3.22]	0.012
No	17 (3.3)	321 (61.7)	1	1	

## Abbreviations

AFB: Acid-Fast Bacilli
AOR: Adjusted odds ratio
CI: Confidence interval
COR: Crude odds ratio
DM: Diabetes mellitus
HIV/AIDS: Human immune virus/acquired immune deficiency syndrome
MDR-TB: Multidrug resistance-tuberculosis
OR: Odds ratio
SPSS: Statistical Packages for Social Sciences
TB: Tuberculosis
WHO: World Health Organization.
